# Study of quality of life and its correlated factors in patients after lumbar fusion for lumbar degenerative disc disease

**DOI:** 10.3389/fsurg.2022.939591

**Published:** 2023-01-06

**Authors:** Meng Chen, Da-Yong Peng, Wen-Xiu Hou, Yang Li, Jing-Kun Li, Hao-Xuan Zhang

**Affiliations:** ^1^ Department of Orthopedic Surgery, The First Affiliated Hospital of Shandong First Medical University, Jinan, China; ^2^ Department of Orthopedic Surgery, Shangdong Provincial Qianfoshan Hospital, Shandong University, Jinan, China; ^3^ Department of Spine Surgery, Shandong University Qilu Hospital, Jinan, Shandong, China

**Keywords:** quality of life, lumbar fusion, lumbar degenerative disc disease, social support, correlated factors

## Abstract

**Background:**

In the present work, we aimed to explore the correlated factors of quality of life in patients receiving lumbar fusion for lumbar degenerative disc disease (DDD) in China.

**Methods:**

A total of 180 patients treated with lumbar fusion were included in the present study. Their general demographic characteristics, Visual Analog Scale (VAS) scores, Japanese Orthopedic Association (JOA) scores, Simplified Coping Style Questionnaire (SCSQ), Social Support Questionnaire (SSQ), and Medical Outcomes Study Short Form 36 (MOS SF-36) were collected and evaluated preoperatively and at 1 year postoperatively.

**Results:**

There were significant improvements in scores of VAS, JOA, and quality of life of patients from preoperation to 1-year postoperation after lumbar fusion. Marital status, with or without children, education level, economic pressure, and social support had significant predictive effects on the physical health of patients undergoing lumbar fusion. Marital status, education level, and economic pressure had significant predictive effects on the mental health of patients undergoing lumbar fusion.

**Conclusions:**

Factors correlated with the physical health of patients after lumbar fusion included positive coping style, negative coping style, social support, age, education level (high school college), disease duration (5–10), suffering from other diseases (combined with two or more other disease) and the number of surgical segments (double and three or more). Factors correlated with the mental health included negative coping style, social support, age, education level (middle school and high school college) and the number of surgical segments (double and three or more). The results verify that these factors were correlated to the patient’s quality of life after lumbar fusion. Emphasizing and selectively intervening these correlated factors can further improve the quality of life in patients receiving lumbar fusion for lumbar degenerative disc disease.

## Introduction

Lumbar fusion has become a standard surgical treatment for a variety of lumbar disorders, and it is widely used in the treatment of lumbar degenerative disc disease (DDD) (1–3). However, the research on the quality of life of patients with lumbar fusion has not received enough attention at present.

With the continuous development of lumbar fusion-related technical progress, promoting humanistic spirit, taking patients as the center, understanding the correlated factors of postoperative quality of life in patients with lumbar fusion, and improving the abilities of the daily life of patients after surgery have become the common concern of the majority of medical workers (4).

To explore the correlated factors of quality of life in patients receiving lumbar fusion for lumbar DDD in China, we included lumbar DDD patients who were treated by lumbar fusion at four medical centers in the present study. We comprehensively determined the quality of life of patients after lumbar fusion, analyzed the relationship of the patient's general demographic characteristics, neural function recovery, coping style, social support and quality of life. This study might provide a new theoretical basis for health care after lumbar fusion to achieve better operation results and maximize the quality of life of patients after lumbar fusion.

## Materials and methods

### Ethics

This study was approved by Medical Ethical Committee of The First Affiliated Hospital of Shandong First Medical University, and it has been registered at http://www.chictr.org.cn/(ChiCTR1800014272). Written informed consent was obtained from all individual patients before enrollment. The identity of each patient was kept confidential.

### Study design

#### Selection and description of participants

Patients aged 50–85 years (67.38 ± 8.15) were enrolled in the present work. The inclusion criteria were as follows: (1) patients with lumbar DDD supported by clinical symptoms as well as imaging data, and (2) patients with no significant improvement after at least 6 months of conservative treatment before surgery. Patients were excluded if they had a spinal tumor, infection, and neurological diseases, such as Alzheimer's disease.

**Figure 1:**
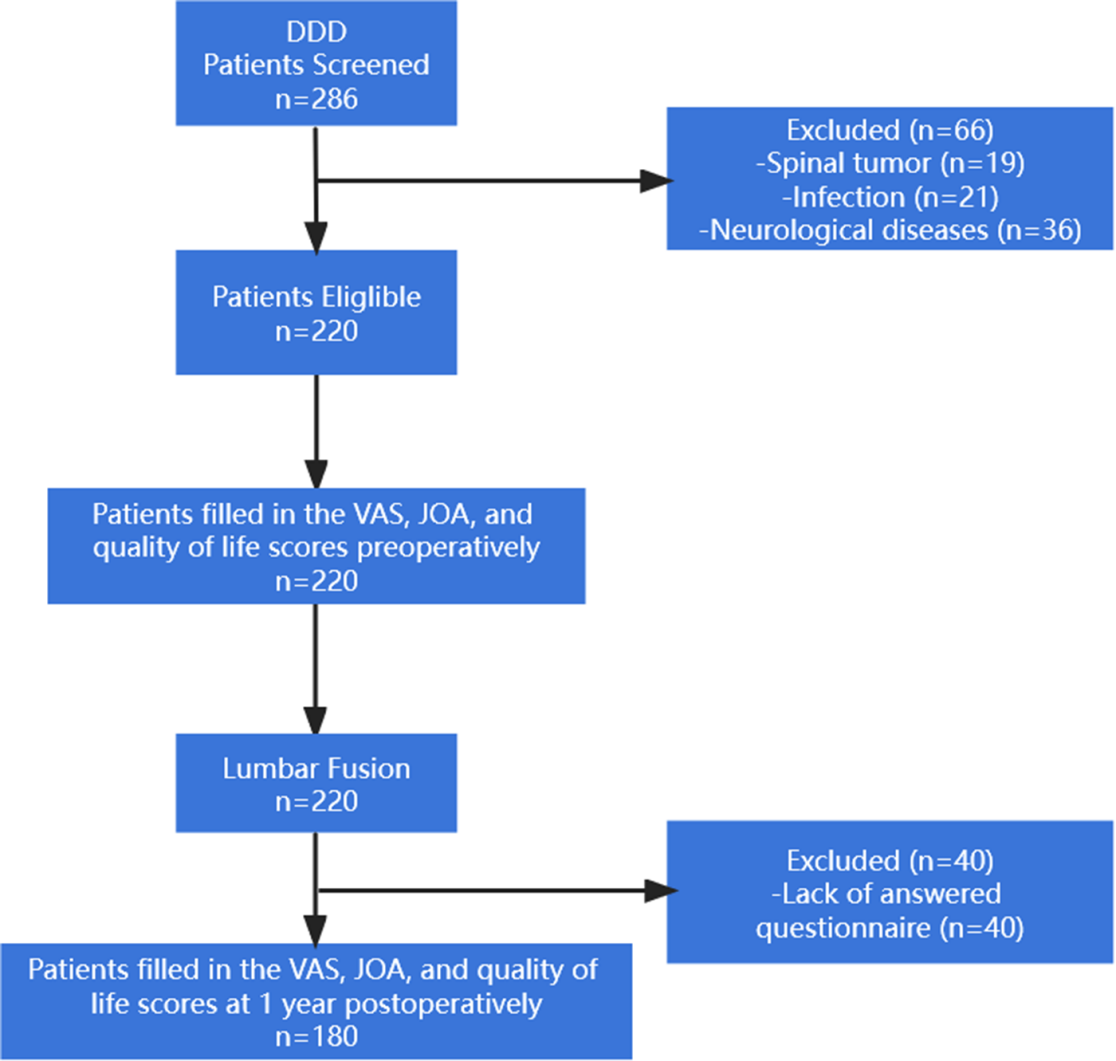
Flowchart showing the patient selection process for this study.

From March 2016 to March 2020, a total of 286 patients with lumbar DDD were identified from four medical institutions across China. 66 not meeting inclusion criteria and 220 patients were treated with lumbar fusion. Among these patients, 132 patients were treated by transforaminal lumbar interbody fusion (TLIF), 77 patients were treated by posterior lumbar interbody fusion (PLIF), and 11 patients were treated by oblique lateral interbody fusion (OLIF). The general demographic characteristics, neurological and functional recovery, coping style, social support, and quality of life of patients were collected preoperatively and at 1 year postoperatively. 40 operated patients were excluded from this study for lack of answered questionnaire. Figure 1 shows the case screening flowchart.

#### Technical information

This study has been registered at http://www.chictr.org.cn/(ChiCTR1800014272). The general demographic characteristics included age, body mass index (BMI), gender, marital status, with or without children, education level, living status, economic pressure, disease duration, whether suffering from other diseases, and the number of surgical segments. Visual Analogue Scale for Pain (VAS) score (5), Japanese Orthopaedic Association (JOA) score (6), Simplified Coping Style Questionnaire (SCSQ) (7), Social Support Questionnaire (SSQ) (8), and Medical Outcomes Study Short Form 36 (MOS SF-36) (9) were used to determine the clinical outcomes and explore the factors correlated the quality of life in patients treated with lumbar fusion. The patient filled in the VAS, JOA, and quality of life scores preoperatively and at 1 year postoperatively.

### Statistics

SPSS statistical software (version 20.0, Chicago, IL, USA) was used for statistical analyses. We used Shapiro-Wilk test to identify the normality of continuous variables. Normally distributed variables were expressed as mean ± standard deviation. Skewed variables were reported as median and interquartile range (IQR). Single-factor analysis of variance (ANOVA) and independent-samples t-test were used to analyze whether demographic characteristics affected the quality of life. Pearson correlation analysis was used to analyze the correlations between VAS, JOA scores, coping style self-assessment (positive and negative), social support, and SF-36 quality of life. The factors correlated with the quality of life of patients were explored by stepwise multivariate linear regression analysis. *P* < 0.05 was considered statistically significant.

## Results

### Demographic characteristics of patients

Table 1 shows the demographic characteristics of the patients treated with lumbar fusion.

**Table 1:** General demographic data.

	Category	Number of people	Percentage (%)
Gender	Male	36	21.43
	Female	132	78.57
Marital status	Without spouse	62	36.9
	With spouse	106	63.1
With or without children	Without children	34	20.24
	With children	134	79.76
Education level	Primary school	66	39.29
	Middle school	61	36.31
	High school college	41	24.4
Living status	Solitary	73	43.45
	Living with family members	59	35.12
	Living in nursing home	36	21.43
Economic pressure	Heavier	50	29.76
	Generally	82	48.81
	Lighter	36	21.43
Disease duration	1–5 years	45	26.79
	5–10 years	55	32.74
	More than 10 years	68	40.48
Suffering from other diseases	No other disease	49	29.17
	Combined with one other disease	77	45.83
	Combined with two or more other diseases	42	25.00
Number of surgical segments	Single	73	43.45
	Double	41	24.4
	Three or more	54	32.14

### The relationship between different demographic characteristics and the quality of life

#### Comparison of VAS scores, JOA scores, and quality of life scores before and after lumbar fusion

The average VAS score before lumbar fusion was 7.27 ± 0.89, and it was decreased to 2.21 ± 1.06 at 1 year after lumbar fusion. The average JOA score before lumbar fusion was 7.56 ± 1.79, and it was increased to 19.48 ± 2.69 at 1 year after lumbar fusion. The postoperative VAS and JOA scores were significantly improved compared with their preoperative scores.

According to the MOS SF-36, the patients' quality of life included physical functioning (PF), physical role (RP), bodily pain (PA), general health status (GH), vitality (VT), social functioning (SF), emotional role (ER) and mental health (SM). The mean scores of these eight aspects were significant improvements from preoperative to postoperative 1 year. The results were expressed in Table 2.

**Table 2:** Comparison of quality of life scores before and after lumbar fusion according to the MOS SF-36.

	Preoperative	Postoperative	*t*	*P*
PF	42.46 ± 8.19	74.05 ± 7.80	68.04	<0.0001
RP	41.94 ± 8.37	56.17 ± 8.12	29.49	<0.0001
BP	32.29 ± 9.48	69.30 ± 10.22	54.07	<0.0001
GH	42.52 ± 8.72	53.96 ± 8.54	27.43	<0.0001
VT	41.61 ± 8.76	61.43 ± 9.35	37.94	<0.0001
SF	42.41 ± 9.18	73.58 ± 8.73	69.72	<0.0001
RE	41.66 ± 9.17	72.80 ± 10.06	54.46	<0.0001
MH	41.45 ± 9.17	71.29 ± 9.31	55.66	<0.0001

#### Comparison of VAS scores, JOA scores, and quality of life scores after lumbar fusion in patients of different genders

**Figure 2:**
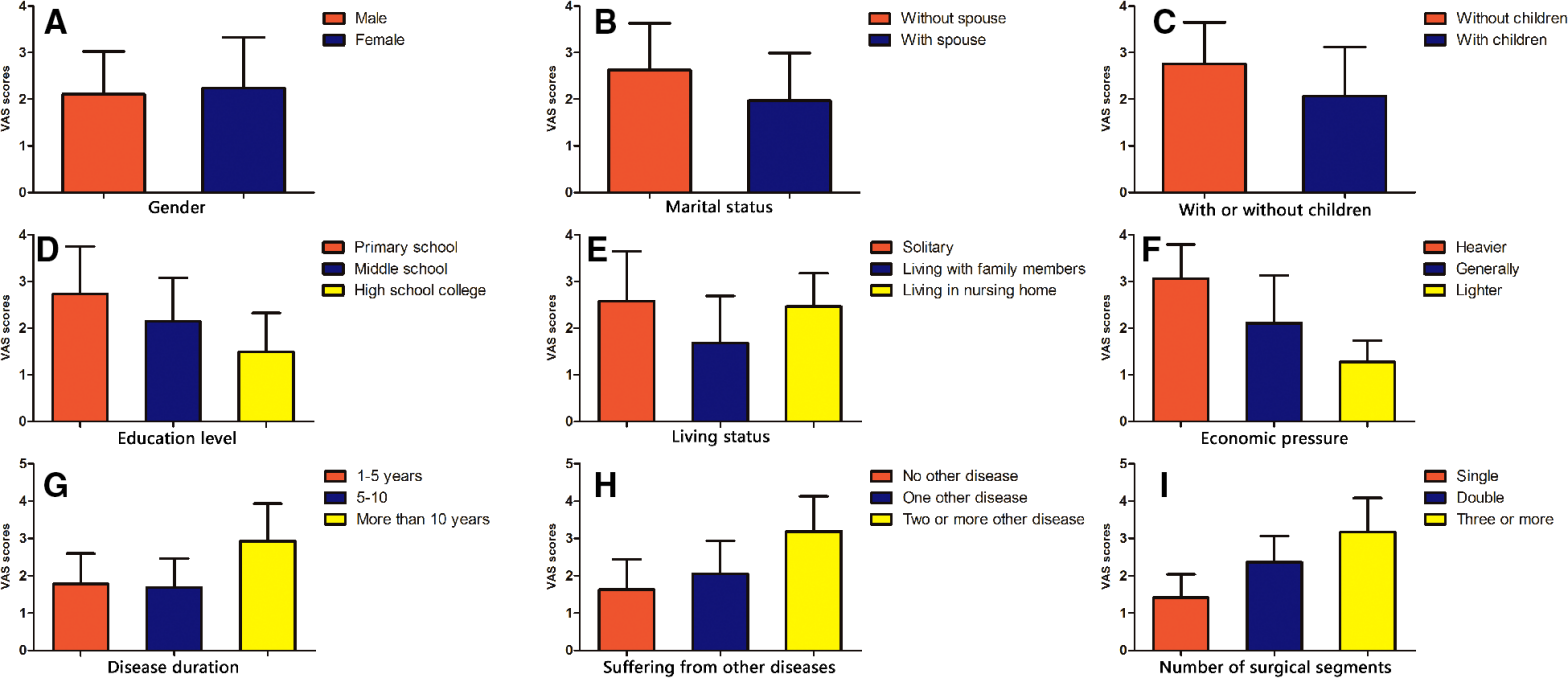
Comparison of VAS scores after lumbar fusion in different demographic characteristics. Comparison of VAS scores after lumbar fusion in different situations, such as gender (**A**), marital status (**B**), with or without children (**C**), education level (**D**), living status (**E**), economic pressure (**F**), disease duration (**G**), suffering from other diseases (**H**) and the number of surgical segments (**I**).

**Figure 3:**
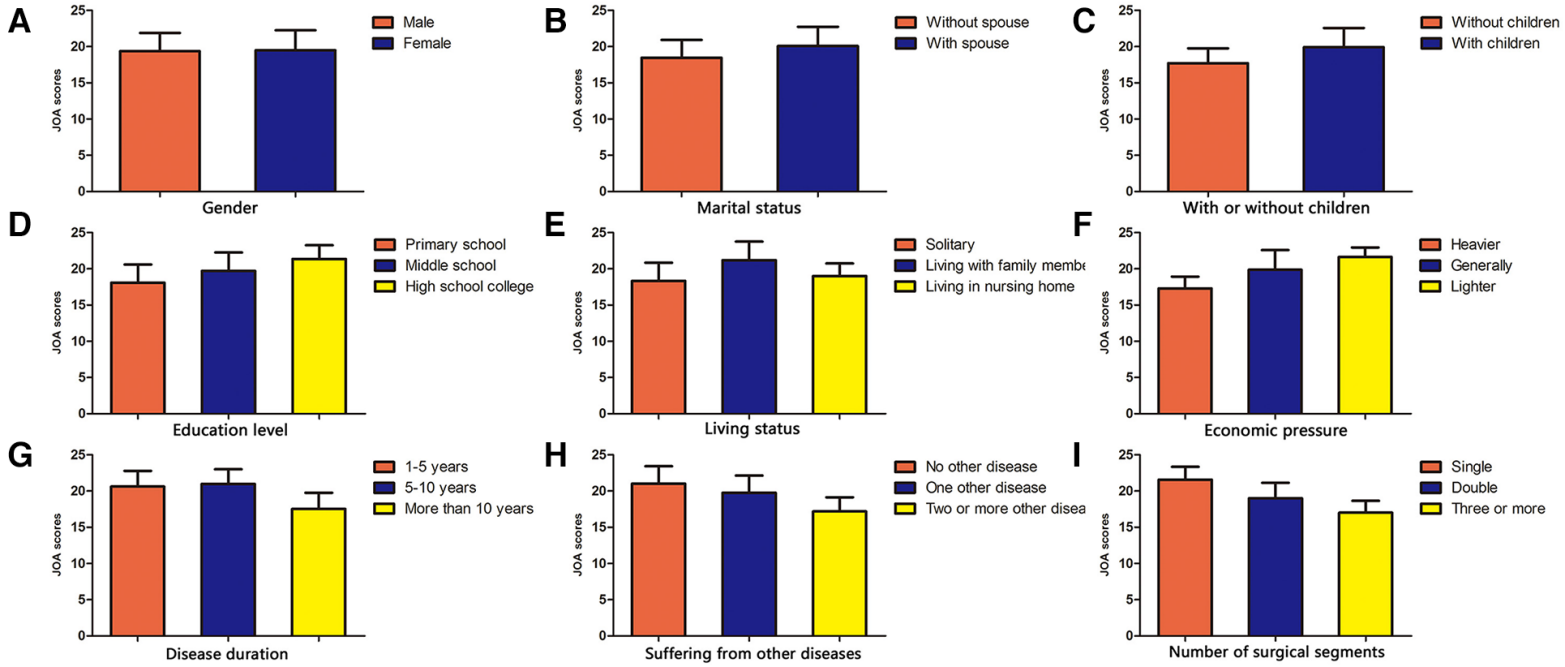
Comparison of JOA scores after lumbar fusion in different demographic characteristics. Comparison of JOA scores after lumbar fusion in different situations, such as gender (**A**), marital status (**B**), with or without children (**C**), education level (**D**), living status (**E**), economic pressure (**F**), disease duration (**G**), suffering from other diseases (**H**) and the number of surgical segments (**I**).

**Figure 4:**
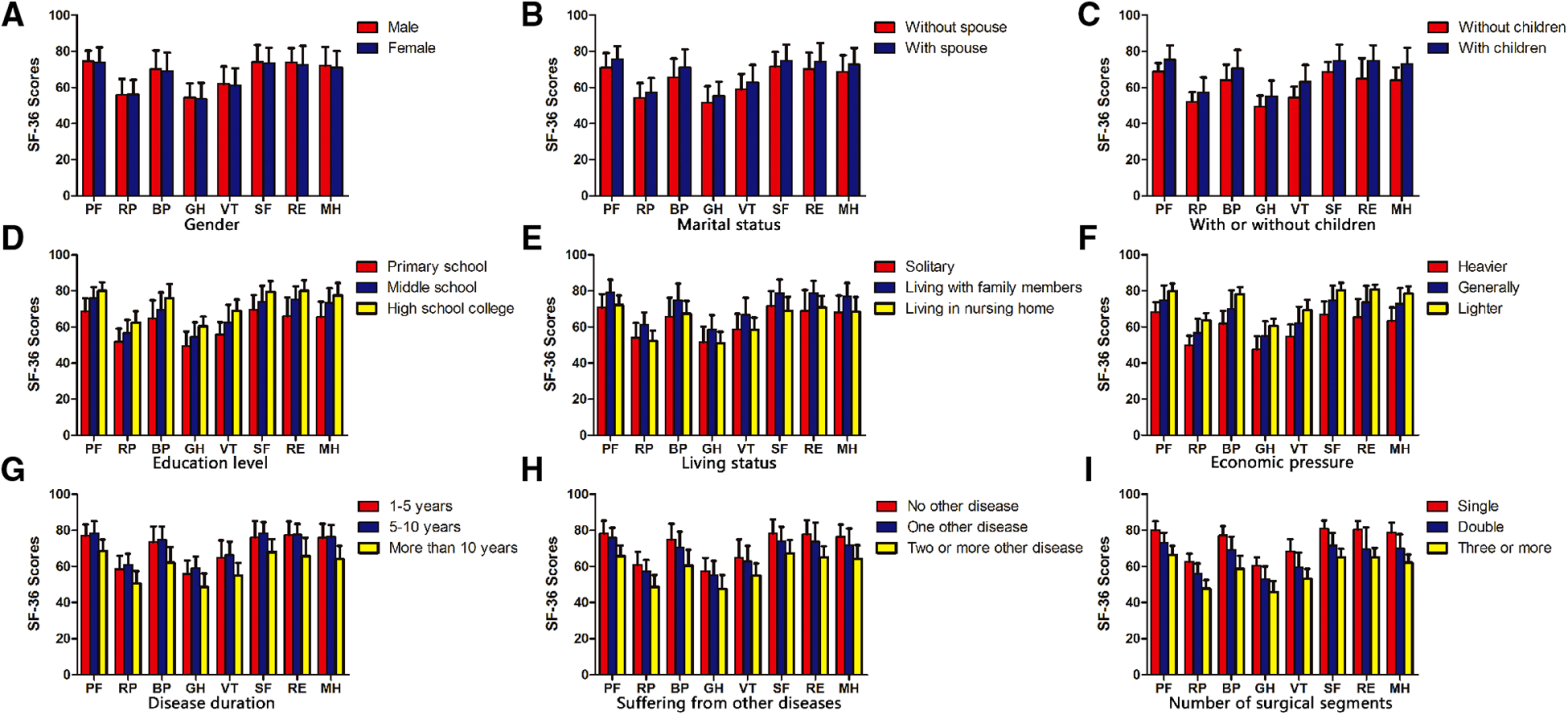
Comparison of quality of life scores after lumbar fusion in different demographic characteristics. Comparison of quality of life scores after lumbar fusion in different situations, such as gender (**A**), marital status (**B**), with or without children (**C**), education level (**D**), living status (**E**), economic pressure (**F**), disease duration (**G**), suffering from other diseases (**H**) and the number of surgical segments (**I**).

The results showed that there was no significant difference in VAS scores, JOA scores, and all eight aspects of the quality of life after lumbar fusion (*P* > 0.05) (Figures 2A, 3A, 4A).

#### Comparison of VAS scores, JOA scores, and quality of life scores after lumbar fusion in patients with different marital statuses

In terms of marital status after lumbar fusion, the VAS scores of unmarried patients were significantly higher compared with those married patients (*P* < 0.05) (Figure 2B). The JOA scores of married patients were significantly higher compared with those unmarried patients (*P* < 0.05) (Figure 3B). In terms of all eight aspects of the quality of life after lumbar fusion, the scores of married patients were significantly higher compared with those unmarried patients (*P* < 0.05) (Figure 4B).

#### Comparison of VAS scores, JOA scores, and quality of life scores after lumbar fusion in patients with or without children

The VAS scores of patients without children were significantly higher compared with those with children (*P* < 0.05) (Figure 2C). The JOA scores of patients with children were significantly higher compared with those without children (*P* < 0.05) (Figure 3C). In terms of all eight aspects of the quality of life after lumbar fusion, the scores of patients with children were significantly higher compared with those without children (*P* < 0.05) (Figure 4C).

#### Comparison of VAS scores, JOA scores, and quality of life scores after lumbar fusion in patients with different education levels

In terms of education level, the higher the degree of the patient's education level, the lower the VAS score (*P* < 0.05) (Figure 2D), and the higher the scores of JOA and all eight aspects of the quality of life (*P* < 0.05) (Figures 3D, 4D).

#### Comparison of VAS scores, JOA scores, and quality of life scores after lumbar fusion in patients different living statuses

In terms of living status after lumbar fusion, the VAS scores of patients living with family members were significantly lower compared with the nursing home group and solitary group (*P* < 0.05), while there was no significant difference between the nursing home group and solitary group (Figure 2E). The scores of JOA and all aspects of the quality of life in patients living with family members were significantly higher compared with the nursing homegroup and solitary group (*P* < 0.05), while there was no significant difference between the nursing home group and solitary group (Figures 3E, 4E).

#### Comparison of VAS scores, JOA scores, and quality of life scores after lumbar fusion in patients with different economic pressures

In terms of economic pressure, the less economic pressure the patient had, the lower the VAS score (*P* < 0.05), and the higher the scores of JOA and all eight aspects of the quality of life (*P* < 0.05) (Figures 2F, 3F, 4F).

#### Comparison of VAS scores, JOA scores, and quality of life scores after lumbar fusion in patients different disease durations

In terms of the patient's disease duration, the VAS scores of the patients in the more than 10-year group were significantly higher compared with the 1–5-year group and 5–10-year group (*p* < 0.05), while there was no significant difference between the 1–5-year group and 5–10-year group (Figure 2G). The scores of JOA and all aspects of the quality of life in the more than 10-year group were significantly lower compared with the 1–5-year group and 5–10-year group (*P* < 0.05), while there was no significant difference between the 1–5-year group and 5–10-year group (Figures 3G, 4G).

#### Comparison of VAS scores, JOA scores, and quality of life scores after lumbar fusion in patients suffering from other diseases

Patients undergoing lumbar fusion may suffer from other diseases, such as coronary heart disease, diabetes, varicose veins of the lower extremities, and so on. In the present study, these patients were divided into three groups as follows: patients with no other disease, patients with one other disease, and patients with two or more other diseases. After lumber fusion, the patients in the no other disease group had the lowest VAS score (*P* < 0.05), followed by patients with one other disease, while the patients with two or more other diseases had the highest score (Figure 2H). Meanwhile, the patients with no other disease had the highest scores of JOA and five aspects of the quality of life (RP, BP, SF, RE, and MH) after lumbar fusion (*P* < 0.05), followed by the patients with one other disease, while the patients with two or more other diseases had the lowest scores (Figures 3H, 4H). In terms of the other three aspects of the quality of life (PF, GH, and VT), the scores of the patients with two or more other diseases were significantly lower compared with the patients with no other disease and those with one other disease (*P* < 0.05), while there was no significant difference between the latter two groups (Figures 3H, 4H).

#### Comparison of VAS scores, JOA scores, and quality of life scores after lumbar fusion in patients with different numbers of surgical segments

Patients undergoing lumbar fusion were divided into three groups according to the number of surgical segments: single-segment group, double-segment group, and multiple-segment groups. The results showed that with the extension of the surgical segment, the postoperative VAS score of patients was higher (*P* < 0.05), and the scores of JOA and all aspects of the quality of life became lower (*P* < 0.05) (Figures 2I, 3I, 4I).

### Correlation between the quality of life and various factors after lumbar fusion

Pearson correlation analysis showed that the positive coping style was positively correlated with the postoperative scores of JOA and all eight aspects of the quality of life, while it was negatively correlated with the postoperative VAS scores (Table 3). The higher the patient's positive coping style, the higher the scores of JOA and all eight aspects of the quality of life after lumbar fusion (*P* < 0.05), while the lower the postoperative VAS scores (*P* < 0.05).

**Table 3:** Correlation between quality of life and various factors after lumber fusion.

	VAS	JOA	PF	RP	BP	GH	VT	SF	RE	MH
Positive coping style										
*r*	−0.55386	0.62106	0.65064	0.57681	0.52797	0.45147	0.45836	0.55274	0.65795	0.63982
*P*	<0.0001	<0.0001	<0.0001	<0.0001	<0.0001	<0.0001	<0.0001	<0.0001	<0.0001	<0.0001
Negative coping style										
*r*	0.73035	−0.67298	−0.78290	−0.74231	−0.74581	−0.69879	−0.68822	−0.75904	−0.70329	−0.84025
*P*	<0.0001	<0.0001	<0.0001	<0.0001	<0.0001	<0.0001	<0.0001	<0.0001	<0.0001	<0.0001
Social support										
*r*	−0.63840	0.78352	0.73882	0.75850	0.70695	0.68580	0.69792	0.75818	0.70920	0.79222
*P*	<0.0001	<0.0001	<0.0001	<0.0001	<0.0001	<0.0001	<0.0001	<0.0001	<0.0001	<0.0001
Age										
*r*	0.52002	−0.52755	−0.45928	−0.52841	−0.52055	−0.45572	−0.39522	−0.45387	−0.37883	−0.40258
*P*	<0.0001	<0.0001	<0.0001	<0.0001	<0.0001	<0.0001	<0.0001	<0.0001	<0.0001	<0.0001
BMI										
*r*	0.55933	−0.50046	−0.58670	−0.51992	−0.52469	−0.51643	−0.48710	−0.56821	−0.55404	−0.60316
*P*	<0.0001	<0.0001	<0.0001	<0.0001	<0.0001	<0.0001	<0.0001	<0.0001	<0.0001	<0.0001

Pearson correlation analysis showed that the negative coping style was positively correlated with the postoperative scores of VAS, while it was negatively correlated with the postoperative scores of JOA and all eight aspects of the quality of life (Table 3). The higher the patient's positive coping style, the higher the postoperative VAS score after lumbar fusion (*P* < 0.05), while the lower the scores of JOA and all eight aspects of the quality of life (*P* < 0.05).

Pearson correlation analysis showed that the patient's social support was positively correlated with the postoperative scores of JOA and all eight aspects of the quality of life, while it was negatively correlated with the postoperative VAS score (Table 3). The higher the patient's social support, the higher the scores of JOA and all eight aspects of the quality of life after lumbar fusion (*P* < 0.05), while the lower the postoperative VAS score (*P* < 0.05).

Pearson correlation analysis showed that the patient's age was positively correlated with the postoperative VAS score, while it was negatively correlated with the postoperative scores of JOA and all eight aspects of the quality of life (Table 3). The older the patient, the higher the postoperative VAS score after lumbar fusion (*P* < 0.05), while the lower the scores of JOA and all eight aspects of the quality of life (*P* < 0.05).

Pearson correlation analysis showed that the patient's BMI was positively correlated with the postoperative VAS score, while it was negatively correlated with the postoperative scores of JOA and all eight aspects of the quality of life (Table 3). The higher the patient's BMI, the higher the postoperative VAS score after lumbar fusion (*P* < 0.05), while the lower the scores of JOA and all eight aspects of the quality of life (*P* < 0.05).

### Multivariate linear stepwise regression analysis of factors correlated with postoperative physical health-related quality of life

PF, RP, BP, and GH in MOS SF-36 are indicators to assess the physical health-related quality of life of a patient. Taking the physical health-related score of SF-36 as a dependent variable, the general demographic data and statistically significant variables in Pearson correlation analysis, which may affect the quality of life after lumbar fusion, were used as independent variables and analyzed by multiple stepwise regression analyses. Multivariate linear stepwise regression analysis screened out nine factors as follows: positive coping style, negative coping style, social support, age, education level (high school college), disease duration (5–10), suffering from other diseases (combined with two or more other disease) and the number of surgical segments (double and three or more) (Table 4). The factors correlated with the physical health-related quality of life after lumbar fusion were positive coping style, negative coping style, social support, age, education level (high school college), disease duration (5–10), suffering from other diseases (combined with two or more other disease) and the number of surgical segments (double and three or more).

**Table 4:** Multivariate linear stepwise regression analysis of factors influencing postoperative physical health-related quality of life.

	B	SE	*β*	*t*	*P*
Positive coping style	−1.080	.457	–0.114	−2.366	0.019
Negative coping style	−3.529	.588	–0.329	−6.003	0.000
Social support	1.711	.552	0.180	3.098	0.002
Age	–0.893	.190	–0.178	−4.708	0.000
Education level (High school college)	9.119	3.401	0.122	2.682	0.008
Disease duration (5–10)	9.793	2.780	0.143	3.522	0.001
Suffering from other diseases (Combined with two or more other disease)	−7.803	3.545	–0.103	−2.201	0.029
The number of surgical segments (Double)	−9.713	3.320	–0.131	−2.926	0.004
The number of surgical segments (Three or more)	−24.764	4.070	–.361	−6.084	0.000

### Multivariate linear stepwise regression analysis of factors correlated with postoperative mental health-related quality of life

VT, SF, RE, and MH in MOS SF-36 are indicators used to assess the mental health-related quality of life of a patient. Taking the mental health-related score of SF-36 as a dependent variable, the general demographic data and statistically significant variables in Pearson correlation analysis, which may affect the quality of life of patients after lumbar fusion, were used as independent variables and analyzed by multiple stepwise regression analysis. Multivariate linear stepwise regression analysis screened out three factors as follows: negative coping style, social support, age, education level (middle school and high school college) and the number of surgical segments (double and three or more) (Table 5). The factors correlated with the mental health-related quality of life of patients after lumbar fusion were negative coping style, social support, age, education level (middle school and high school college) and the number of surgical segments (double and three or more).

**Table 5:** Multivariate linear stepwise regression analysis of factors influencing postoperative mental health-related quality of life.

	B	SE	*β*	*t*	*P*
Negative coping style	−4.554	.654	–.402	−6.961	.000
Social support	2.775	.615	.277	4.515	.000
Age	–.592	.211	–.112	−2.807	.006
Education level (Middle school)	6.396	3.040	.091	2.104	.037
Education level (High school college)	9.641	3.784	.122	2.548	.012
The number of surgical segments (Double)	−7.697	3.694	–.098	−2.083	.039
The number of surgical segments (Three or more)	−12.621	4.530	–.175	−2.786	.006

### Multivariate linear stepwise regression analysis of correlated factors of postoperative VAS scores

Taking the postoperative VAS score as a dependent variable, the general demographic data and statistically significant variables in Pearson correlation analysis, which may affect the quality of life of patients after lumbar fusion, were used as independent variables and analyzed by multiple stepwise regression analysis. Multivariate linear stepwise regression analysis screened out seven factors as follows: negative coping style, age, marital status, education level (middle school), economic pressure (lighter) and the number of surgical segments (double and three or more) (Table 6). The factors correlated with the postoperative VAS score of patients after lumbar fusion included negative coping style, age, marital status, education level (middle school), economic pressure (lighter) and the number of surgical segments (double and three or more).

**Table 6:** Multivariate linear stepwise regression analysis of influencing factors of postoperative VAS score.

	B	SE	*β*	*t*	*P*
Negative coping style	.100	.031	.282	3.220	.002
Age	.028	.010	.167	2.763	.006
Marital status	–.267	.115	–.123	−2.321	.022
Education level (Middle school)	.358	.144	.163	2.479	.014
Economic pressure (Lighter)	–.532	.221	–.208	−2.414	.017
The number of surgical segments (Double)	.592	.175	.241	3.375	.001
The number of surgical segments (Three or more)	.843	.215	.372	3.918	.000

### Multivariate linear stepwise regression analysis of correlated factors of postoperative JOA scores

Taking the postoperative JOA score as a dependent variable, the general demographic data and statistically significant variables in Pearson correlation analysis, which may affect the quality of life of patients after lumbar fusion, were used as independent variables and analyzed by multiple stepwise regression analysis. Multivariate linear stepwise regression analysis screened out six factors as follows: social support, age, living status (living in nursing home), economic pressure (generally and lighter) and the number of surgical segments (three or more) (Table 7). The factors correlated with the postoperative JOA score of patients after lumbar fusion included social support, age, living status (living in nursing home), economic pressure (generally and lighter) and the number of surgical segments (three or more).

**Table 7:** Multivariate linear stepwise regression analysis of influencing factors of postoperative JOA score.

	B	SE	*β*	*t*	*P*
Social support	0.324	0.072	0.400	4.467	0.000
Age	–0.060	0.025	–0.140	−2.402	0.017
Living status (Living in nursing home)	0.839	0.379	0.128	2.216	0.028
Economic pressure (Generally)	0.840	0.383	0.154	2.192	0.030
Economic pressure (Lighter)	1.390	0.548	0.211	2.537	0.012
The number of surgical segments (Three or more)	−1.343	0.534	–0.230	−2.513	0.013

## Discussion

As a classical method for the treatment of lumbar DDD, lumbar fusion has been widely proved to clinically provide stability for lumbar DDD, especially lumbar disc herniation, lumbar spinal stenosis, and spondylolisthesis (10, 11). Although patients have improved quality of life after lumbar fusion (12, 13), the patient's recovery and improvements of postoperative physical, psychological, and social functions are still not satisfied due to the continuous effects of the disease itself, surgical trauma, and other correlated factors (1, 2, 14).

In addition to the treatment of physical diseases, it is an ideal target to focus on the adjustment of patients' mental health, promote the recovery of social life, and improve the quality of life for the treatment of lumbar disorders. Therefore, a comprehensive assessment of the quality of life after lumbar fusion and analysis of its correlated factors will further improve the quality of life of patients after surgery, which is more in line with the humanistic medicine development direction of people-oriented and patient-centered.

### The relationship between different demographic characteristics and the quality of life

#### The correlativeness of demographic characteristics and VAS, JOA, quality of life scores

To explore the factors correlated the quality of life of patients undergoing lumbar fusion for lumbar DDD, demographic data were collected as independent factors. The MOS SF-36 health survey profile was used for comparative analysis.

Specific analysis was conducted as follows:

Significant improvements in the VAS, JOA, and quality of life scores for both males and females were observed. However, there was no significant difference in the quality of life between different genders after lumbar fusion. This finding suggested that gender was not the correlated factor of postoperative quality of life after lumbar fusion, which was also similar to the results of the Kapetanakis study on transforaminal percutaneous endoscopic discectomy for lumbar disc herniation in different genders.

The VAS, JOA, and quality of life scores of married patients were significantly better compared with those unmarried patients, showing that companionship, care, and support in physical rehabilitation and psychological counseling were beneficial to the physical health and mental health of patients, which might help patients integrate into social life smoothly after surgery.

Patients with children had significantly better VAS, JOA, and quality of life scores compared with those without children, suggesting that children played a positive role in improving the quality of life.

The higher the education level of patients, the better the scores of VAS, JOA, and postoperative quality of life. Highly educated patients will increase their knowledge of the disease itself through books, networks, and connections, make physical and psychological preparations, and have good expression and communication skills, which all play a certain role in improving the physical, mental health, and social life status of patients after lumbar fusion. However, patients with low educational backgrounds have relatively weak health awareness and limited ability to obtain medical assistance, thus affecting their quality of life after lumbar fusion.

The VAS, JOA, and quality of life scores of patients living with the family were significantly better compared with the solitary group and nursing home group. This result reflected that family members paid more attention to the patient's postoperative recovery and improvement, and helped patients integrate into society more quickly. However, the patients in the solitary group and nursing home group relatively lacked the related assistance measures. Peeters has found that optimizing nutrition intake, (home) rehabilitation programs, and the possibility for psychological counseling contribute to postoperative rehabilitation in patients after hip fracture surgery (15).

The greater the patient's economic pressure, the worse the VAS, JOA, and quality of life scores of patients after lumbar fusion. Patients with better economic conditions meant less economic and psychological burden, and there would be better functional and psychological recovery conditions after surgery, resulting in a greater improvement in the quality of life. This finding was consistent with the results of McCabew on the impact of economic burden on quality of life (16).

Compared with patients with a disease duration of shorter than 5 years and 5–10 years, patients with a disease duration of more than 10 years had poorer VAS, JOA, and quality of life scores. This finding indicated that patients with shorter disease duration and less severe illness were more satisfied with the improvement of postoperative quality of life.

Patients with fewer other diseases had significantly better VAS, JOA, and quality of life scores than those with two or more other diseases, such as coronary heart disease, diabetes, varicose veins of the lower extremities, and so on. Other diseases increase the risk of perioperative complications and harm the recovery of postoperative quality of life (17).

The fewer the surgical segments of the patient, the better the postoperative VAS, JOA, and quality of life scores. The number of surgical segments is related to the length of time of the surgery and the degree of iatrogenic injury, which may affect the patient's physical condition and mental state, exerting a corresponding impact on the recovery of postoperative quality of life.

### Correlation between quality of life and various factors after lumbar fusion

The Pearson correlation analysis showed that the positive coping style, social support, and BMI of patients were positively correlated with postoperative JOA and quality of life scores, while these factors were negatively correlated with postoperative VAS scores. On the contrary, the negative coping style, age, and BMI of patients were positively correlated with postoperative VAS scores, while they were negatively correlated with postoperative JOA and quality of life scores.

The patient can obtain satisfactory postoperative functional recovery, physical, and mental health through using a positive attitude to adjust mental state after surgery and the social support provided by family members and friends, thus achieving an ideal quality of life after lumbar fusion (18, 19). However, if the patient adopts a negative mentality to deal with postoperative recovery and life after surgery, it will have a heavy psychological burden, which is not conducive to postoperative physical and psychological recovery and will not be able to obtain a satisfactory quality of life. Therefore, medical staff should recognize the impact of personal coping style and social support on the quality of life of patients, and actively improve the quality of life of patients by improving these aspects.

The older the patient, the lower the postoperative scores of VAS, JOA, and quality of life. This finding showed that with the increase of age, the patient's body function was gradually reduced, making it less tolerant to postoperative rehabilitation training, which in turn affected the effect of surgery. Meanwhile, older patients tended to be in a state of retirement, and mental life was increasingly scarce, which also affected their quality of life.

The higher the BMI, the lower the postoperative scores of VAS, JOA, and quality of life. Obesity itself is one of the risk factors for lumbar-related diseases (20). A high BMI will increase the burden on the lumbar spine, which will harm the postoperative functional exercise and living standards of patients, and also affect the quality of life of patients after lumbar fusion.

### Multivariate regression analysis of factors correlated with the quality of life in patients after lumbar fusion

#### Analysis of correlated factors of patients' physical health

Multivariate stepwise regression analysis showed that positive coping style, negative coping style, social support, age, education level (high school college), disease duration (5–10), suffering from other diseases (combined with two or more other disease) and the number of surgical segments (double and three or more) had significant predictive effects on the physical health of patients undergoing lumbar fusion.

Positive coping style, social support and younger age are protective factors for the postoperative physical health of patients. The social support provided by family members and friends play an extremely important role in the recovery process after lumbar fusion. They can provide a comfortable living environment, supervise patients' rehabilitation training, communicate with patients, and provide high social support to promote the formation and maintenance of positive coping style as well as the comprehensive recovery of physical function, psychological and social life, thus improving the quality of life of patients (21). On the contrary, many patients may suffer from muscle pain, irregular rehabilitation training, and poor exercise compliance due to negative coping style, older age, or even lack of care for their family members. In addition, the communication and coordination ability of this group of people is relatively insufficient, which will cause unsatisfactory recovery after surgery, affecting the patient's sleep quality, daily life, and social life, and aggravating the psychological burden, as well as the physical and physiological aspects of the patient. These negative effects will further affect the physical function recovery, mental health, and social activity status of patients, resulting in a decline in the quality of life of patients (22).

Patients with a high degree of education will take the initiative to understand the relevant knowledge of the disease. They can be exposed to scientific postoperative rehabilitation methods through various media, their compliance with physician guidance is high, and these all play an active role in improving the physical health of patients after surgery.

Disease duration (5–10), suffering from other diseases (combined with two or more other disease) and the number of surgical segments (double and three or more) were the factors correlating the physical health of patients after lumbar fusion. These often meant that patients were in poorer physical condition and had a more severe condition, which could not conducive to the recovery of physiological functions of the body, and the process of postoperative recovery of the body function was relatively hard.

#### Analysis of factors correlated with patients' mental health

Multivariate stepwise regression analysis showed that negative coping style, social support, age, education level (middle school and high school college) and the number of surgical segments (double and three or more) had significant predictive effects on the mental health of patients undergoing lumbar fusion.

Patients can get social support such as material support and spiritual comfort from their family members and friends, which can help alleviate the psychological burden of patients, restore their social life more quickly, and actively promote the physical and mental recovery of patients after surgery.

A higher level of education can promote the mental health of patients after lumbar fusion. This may be attributed to that people with higher education have more ways to obtain spiritual satisfaction, and their mental and mental adjustment, social adaptation, and coordination are stronger, which is conducive to the adjustment and recovery of postoperative psychological state (23).

Patients with negative coping style, advanced age, multi-segment fusion have relatively poor living and functional exercise conditions after surgery, and they are unwilling to continue functional exercise due to pain in the surgical site and temporary physical activity disorder, forming a vicious circle of functional exercise. These will bring tremendous psychological pressure to the patients and produce negative emotions, such as anxiety, resistance, and depression, which is not conducive to the recovery of postoperative physical and mental health and improvement of quality of life (24).

### Multivariate linear stepwise regression analysis of correlated factors of postoperative VAS/JOA scores

Multivariate stepwise regression analysis showed that the factors correlated with the VAS score of patients after lumbar fusion were negative coping style, age, marital status, education level (middle school), economic pressure (lighter) and the number of surgical segments (double and three or more). Postoperative VAS scores represent subjective perceptions of postoperative pain relief in patients. Negative coping style, advanced age, marital status without spouse, too high or too low educational level, high economic pressure and multi-segment fusion are not conducive to the improvement of postoperative pain relief. The factors correlated with the JOA score of patients after lumbar fusion were social support, age, living status (living in nursing home), economic pressure (generally and lighter) and the number of surgical segments (three or more). The postoperative JOA scores reflect the improvement of lumbar function before and after surgery. Insufficient social support, advanced age, solitary, living with family members, high economic pressure, and multi-segment fusion affect the therapeutic effect of lumbar fusion, which is not conducive to the improvement of postoperative lumbar function.

## Conclusions

Factors correlated with the physical health-related quality of life after lumbar fusion included positive coping style, negative coping style, social support, age, education level (high school college), disease duration (5–10), suffering from other diseases (combined with two or more other disease) and the number of surgical segments (double and three or more). Factors correlated with mental health were negative coping style, social support, age, education level (middle school and high school college) and the number of surgical segments (double and three or more). The correlated factors of VAS scores in patients after lumbar fusion were negative coping style, age, marital status, education level (middle school), economic pressure (lighter), the number of surgical segments (double and three or more) and the factors affecting JOA scores were social support, age, living status (living in nursing home), economic pressure (generally and lighter) and the number of surgical segments (three or more).

Collectively, these results confirmed that these factors were correlated to the quality of life in patients receiving lumbar fusion for lumbar degenerative disc disease. Emphasizing and selectively intervening these correlated factors can help to provide targeted individualized psychological counseling and rehabilitation education for postoperative patients while providing scientific and effective family support and social support, which is conducive to maximizing the therapeutic effect of surgery and improving the quality of life in patients receiving lumbar fusion for lumbar degenerative disc disease.

## Data Availability

The original contributions presented in the study are included in the article/Supplementary Materials, further inquiries can be directed to the corresponding author/s.

## References

[1] Divi SN Schroeder GD Goyal DKC Radcliff KE Galetta MS Hilibrand AS Fusion technique does not affect short-term patient-reported outcomes for lumbar degenerative disease. Spine J. (2019) 19(12):1960–8. 10.1016/j.spinee.2019.07.014 31356987

[2] Jin M Zhang J Shao H Liu J Huang Y. Percutaneous transforaminal endoscopic lumbar interbody fusion for degenerative lumbar diseases: a consecutive case series with mean 2-year follow-up. Pain Physician. (2020) 23(2):165–74. 10.36076/ppj.2020/23/165 32214300

[3] Driver J Huang KT Krag M Bydon M Nunley P Lavoie S One year outcomes from a prospective multicenter investigation device trial of a novel conformal mesh interbody fusion device. Spine. (2021) 46(2):E126–E132. 10.1097/BRS.0000000000003710 32991515

[4] Pierce KE Passias PG Alas H Brown AE Bortz CA Lafage R International spine study group (ISSG). does patient frailty status influence recovery following spinal fusion for adult spinal deformity?: an analysis of patients with 3-year follow-up. Spine. (2020) 45(7):E397–405. 10.1097/BRS.0000000000003288 31651683

[5] Divi SN Goyal DKC Galetta MS Fang T Padua FG Reyes AA How does body mass index influence outcomes in patients after lumbar fusion? Spine. (2020) 45(8):555–61. 10.1097/BRS.0000000000003313 31770335

[6] Masuda K Higashi T Yamada K Sekiya T Saito T. The surgical outcome of decompression alone versus decompression with limited fusion for degenerative lumbar scoliosis. J Neurosurg Spine. (2018) 29(3):259–64. 10.3171/2018.1.SPINE17879 29856301

[7] Liao H Tang W Huang Y Liu M Zhang Y Zhang L Stressors, coping styles, and anxiety & depression in pediatric nurses with different lengths of service in six tertiary hospitals in Chengdu, China. Transl Pediatr. (2020) 9(6):827–34. 10.21037/tp-20-439 33457305 PMC7804478

[8] Kroplewski Z Serocka A Szcześniak M. Social support and sense of life in patients with anxiety disorders-preliminary report. Psychiatr Pol. (2019) 53(2):313–24. 10.12740/PP/81447 31317960

[9] Pozza A Osborne RH Elsworth GR Gualtieri G Ferretti F Coluccia A. Evaluation of the health education impact questionnaire (heiQ), a self-management skill assessment tool, in Italian chronic patients. Psychol Res Behav Manag. (2020) 13:459–71. 10.2147/PRBM.S245063 32547268 PMC7246315

[10] Ding F Jia Z Zhao Z Xie L Gao X Ma D Total disc replacement versus fusion for lumbar degenerative disc disease: a systematic review of overlapping meta-analyses. Eur Spine J. (2017) 26(3):806–15. 10.1007/s00586-016-4714-y 27448810

[11] Momin AA Barksdale EM 3rd Lone Z Enders JJ Nowacki AS Winkelman RD Exploring perioperative complications of anterior lumber interbody fusion in patients with a history of prior abdominal surgery: a retrospective cohort study. Spine J. (2020) 20(7):1037–43. 10.1016/j.spinee.2020.03.009 32200118

[12] Makino T Kaito T Fujiwara H Honda H Sakai Y Takenaka S Risk factors for poor patient-reported quality of life outcomes after posterior lumbar interbody fusion: an analysis of 2-year follow-up. Spine. (2017) 42(19):1502–10. 10.1097/BRS.0000000000002137 28248893

[13] Soliman HAG Barchi S Parent S Maurais G Jodoin A Mac-Thiong JM. Early impact of postoperative bracing on pain and quality of life after posterior instrumented fusion for lumbar degenerative conditions: a randomized trial. Spine. (2018) 43(3):155–60. 10.1097/BRS.0000000000002292 28632643

[14] Jain D Berven SH Carter J Zhang AL Deviren V. Bariatric surgery before elective posterior lumbar fusion is associated with reduced medical complications and infection. Spine J. (2018) 18(9):1526–32. 10.1016/j.spinee.2018.01.023 29408400

[15] Peeters CM Visser E Van de Ree CL Gosens T Den Oudsten BL De Vries J. Quality of life after hip fracture in the elderly: a systematic literature review. Injury. (2016) 47(7):1369–82. 10.1016/j.injury.2016.04.018 27178770

[16] McCabew MP De Judicibus M. The effects of economic disadvantage on psychological well-being and quality of life among people with multiple sclerosis. J Health Psychol. (2005) 10(1):163–73. 10.1177/1359105305048562 15576507

[17] Amaral R Ferreira R Marchi L Jensen R Nogueira-Neto J Pimenta L. Stand-alone anterior lumbar interbody fusion-complications and perioperative results. Rev Bras Ortop. (2017) 52(5):569–74. 10.1016/j.rboe.2017.08.016 29062822 PMC5643906

[18] Pozuelo-Carrascosa DP Martínez-Vizcaíno V Sánchez-López M Bartolomé-Gutiérrez R Rodríguez-Martín B Notario-Pacheco B. Resilience as a mediator between cardiorespiratory fitness and mental health-related quality of life: a cross-sectional study. Nurs Health Sci. (2017) 19(3):316–21. 10.1111/nhs.12347 28590081

[19] Rahman S Mittendorfer-Rutz E Dorner TE Pazarlis K Ropponen A Svedberg P Work-disability in low back pain patients with or without surgery, and the role of social insurance regulation changes in Sweden. Eur J Public Health. (2019) 29(3):524–30. 10.1093/eurpub/cky243 30445623

[20] Khan JM Basques BA Kunze KN Grewal G Hong YS Pardo C Does obesity impact lumbar sagittal alignment and clinical outcomes after a posterior lumbar spine fusion? Eur Spine J. (2020) 29(2):340–8. 10.1007/s00586-019-06094-y 31420726

[21] Ullrich A Ascherfeld L Marx G Bokemeyer C Bergelt C Oechsle K. Quality of life, psychological burden, needs, and satisfaction during specialized inpatient palliative care in family caregivers of advanced cancer patients. BMC Palliat Care. (2017) 16(1):31. 10.1186/s12904-017-0206-z 28486962 PMC5424283

[22] Stanley S Balakrishnan S Ilangovan S. Psychological distress, perceived burden and quality of life in caregivers of persons with schizophrenia. J Ment Health. (2017) 26(2):134–41. 10.1080/09638237.2016.1276537 28385096

[23] Schandl AR Johar A Mälberg K Lagergren P. Education level and health-related quality of life after oesophageal cancer surgery: a nationwide cohort study. BMJ Open. (2018) 8(8):e020702. 10.1136/bmjopen-2017-020702 30139895 PMC6112400

[24] Hsiao FH Kuo WH Jow GM Wang MY Chang KJ Lai YM The changes of quality of life and their correlations with psychosocial factors following surgery among women with breast cancer from the post-surgery to post-treatment survivorship. Breast. (2019) 44:59–65. 10.1016/j.breast.2018.12.011 30669032

