# Corrigendum: Study of quality of life and its correlated factors in patients after lumbar fusion for lumbar degenerative disc disease

**DOI:** 10.3389/fsurg.2024.1371641

**Published:** 2024-02-15

**Authors:** Meng Chen, Da-Yong Peng, Wen-Xiu Hou, Yang Li, Jing-Kun Li, Hao-Xuan Zhang

**Affiliations:** ^1^ Department of Orthopedic Surgery, The First Affiliated Hospital of Shandong First Medical University, Jinan, China; ^2^ Department of Orthopedic Surgery, Shangdong Provincial Qianfoshan Hospital, Shandong University, Jinan, China; ^3^ Department of Spine Surgery, Shandong University Qilu Hospital, Jinan, Shandong, China

**Keywords:** quality of life, lumbar fusion, lumbar degenerative disc disease, social support, correlated factors

A Corrigendum on Study of quality of life and its correlated factors in patients after lumbar fusion for lumbar degenerative disc disease By Chen M, Peng D-Y, Hou W-X, Li Y, Li J-K, Zhang H-X. 2023 Front. Surg. 9:939591. doi: 10.3389/fsurg.2022.939591

Error in Figure

In the published article, there was an error in Figure 3 as published. An error occurred during the correction phase of the publication. Figure 3 should be Comparison of JOA (NOT VAS) scores after lumbar fusion in different demographic characteristics, and the figure legend is correct. The corrected Figure 3 and its caption “Comparison of JOA scores after lumbar fusion in different demographic characteristics” appear below.

**Figure 3:**
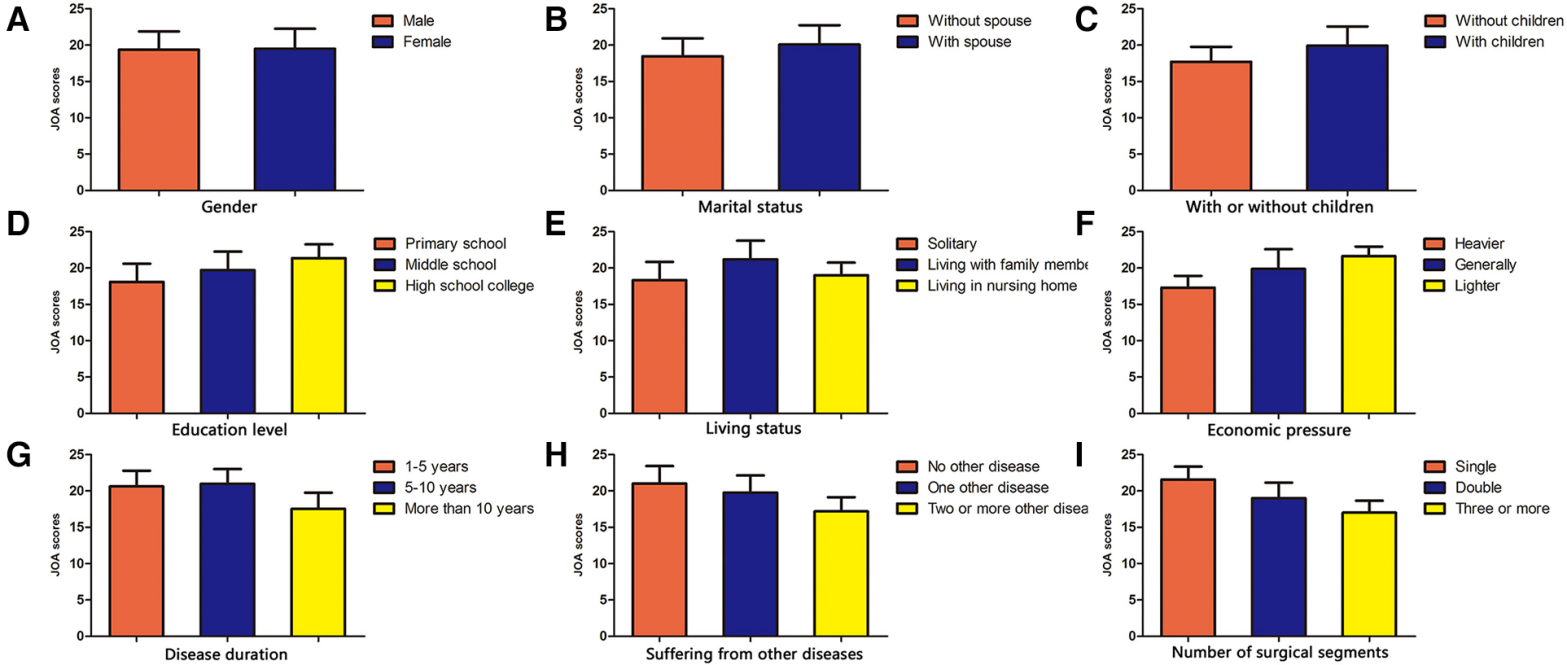
Comparison of JOA scores after lumbar fusion in different demographic characteristics.

The authors apologize for this error and state that this does not change the scientific conclusions of the article in any way. The original article has been updated.

## Publisher's note

All claims expressed in this article are solely those of the authors and do not necessarily represent those of their affiliated organizations, or those of the publisher, the editors and the reviewers. Any product that may be evaluated in this article, or claim that may be made by its manufacturer, is not guaranteed or endorsed by the publisher.

